# Swi1 Associates with Chromatin through the DDT Domain and Recruits Swi3 to Preserve Genomic Integrity

**DOI:** 10.1371/journal.pone.0043988

**Published:** 2012-08-30

**Authors:** Chiaki Noguchi, Jordan B. Rapp, Yuliya V. Skorobogatko, Lauren D. Bailey, Eishi Noguchi

**Affiliations:** Department of Biochemistry and Molecular Biology, Drexel University College of Medicine, Philadelphia, Pennsylvania, United States of America; University of Minnesota, United States of America

## Abstract

Swi1 and Swi3 form the replication fork protection complex and play critical roles in proper activation of the replication checkpoint and stabilization of replication forks in the fission yeast *Schizosaccharomyces pombe*. However, the mechanisms by which the Swi1-Swi3 complex regulates these processes are not well understood. Here, we report functional analyses of the Swi1-Swi3 complex in fission yeast. Swi1 possesses the DDT domain, a putative DNA binding domain found in a variety of chromatin remodeling factors. Consistently, the DDT domain-containing region of Swi1 interacts with DNA in vitro, and mutations in the DDT domain eliminate the association of Swi1 with chromatin in *S. pombe* cells. DDT domain mutations also render cells highly sensitive to S-phase stressing agents and induce strong accumulation of Rad22-DNA repair foci, indicating that the DDT domain is involved in the activity of the Swi1-Swi3 complex. Interestingly, DDT domain mutations also abolish Swi1’s ability to interact with Swi3 in cells. Furthermore, we show that Swi1 is required for efficient chromatin association of Swi3 and that the Swi1 C-terminal domain directly interacts with Swi3. These results indicate that Swi1 associates with chromatin through its DDT domain and recruits Swi3 to function together as the replication fork protection complex.

## Introduction

In response to replication stress, cells activate the DNA replication checkpoint to arrest the cell cycle and allow time for DNA repair. Central to this system are protein kinases such as human ATM and ATR, fission yeast Rad3, and budding yeast Mec1 [1], [2], [3], [4], [5]. These kinases are required for activation of downstream effector kinases by phosphorylation. In fission yeast, Rad3 activates Cds1 and Chk1 kinases in response to replication stress or DNA damage, facilitating DNA repair and recombination pathways [1], [3], [6]. Another essential function of the replication checkpoint is to stabilize replication forks by maintaining proper assembly of replisome components and preserving DNA structures during DNA replication problems [7], [8], [9], [10], [11]. Recent studies found that ancillary factors, which are not essential for DNA synthesis but are important for DNA replication accuracy, also travel with moving replication forks. Such factors include fission yeast Swi1 and Swi3, which together form the replication fork protection complex (FPC) and are required for efficient activation of the replication checkpoint kinase Cds1 and stabilization of stalled replication forks [12], [13], [14]. In the absence of Swi1 or Swi3, cells accumulate abnormal fork structures that lead to Rad22 DNA repair foci formation and accumulation of recombination structures during S phase [13], [15]. It has also been shown that the Swi1-Swi3 complex directly interacts with DNA and recruits Mrc1, a mediator of the replication checkpoint, to the replication fork [16], [17]. Furthermore, genetic studies in yeast suggest that the Swi1-Swi3 FPC has roles in coordinating leading- and lagging-strand DNA synthesis and in coupling DNA polymerase and helicase activities at the replication fork [12], [13], [18]. In addition, Swi1 and Swi3 are involved in the establishment of sister chromatid cohesion at the replication fork [19], suggesting the importance of Swi1-Swi3 in coordinating multiple cellular events at the replication forks. The functions of the Swi1-Swi3 complex are conserved among eukaryotes [12], [20], [21], [22]. Studies show that Swi1-Swi3 orthologues (Tof1-Csm3 in budding yeast, and Timeless-Tipin in vertebrates, respectively) are components of the replisome and that they are involved in fork stabilization, the intra-S phase checkpoint, and the establishment of sister chromatid cohesion [12], [20], [23], [24], [25], [26], [27], [28], [29], [30]. However, how the FPC protects replication forks and coordinates with multiple genome maintenance processes at the replication fork is not well understood.

In our previous studies, we have reported separation-of-function mutants of Swi3 and dissected the molecular pathways that require Swi1-Swi3 functions [31]. Our investigation demonstrated that Swi3 activates two separate pathways to promote replication fork recovery in response to different genotoxic agents. Swi3 promotes efficient restart of stalled replication forks in a checkpoint-dependent manner. However, Swi3 restores broken replication forks in a checkpoint-independent manner, which appeared to be coupled with the establishment of sister chromatid cohesion. In addition, we demonstrated that Swi1-Swi3 complex formation is necessary for its functions in genome maintenance mechanisms [31]. However, the molecular basis of Swi1-Swi3 complex formation and its chromatin association remains elusive. Therefore, in this study, we have carried out domain analyses of Swi1 to understand the mechanisms by which the Swi1-Swi3 complex preserves genomic integrity. We describe Swi1 domains that are required for its chromatin association and Swi1-Swi3 complex formation. Interestingly, we found that Swi1 possesses the DDT domain, a putative DNA binding domain that is often found in chromatin associating factors [32]. We show that the DDT domain of Swi1 is involved in its association with chromatin and efficient recruitment of Swi3 to chromatin. Consistently, DDT domain mutants show hypersensitivity to genotoxic agents and accumulate DNA damage. These data will provide important insights into understanding Swi1-Swi3-dependent replication fork stabilization and checkpoint activation.

## Results

### Structural Prediction of Swi1

**Figure 1:**
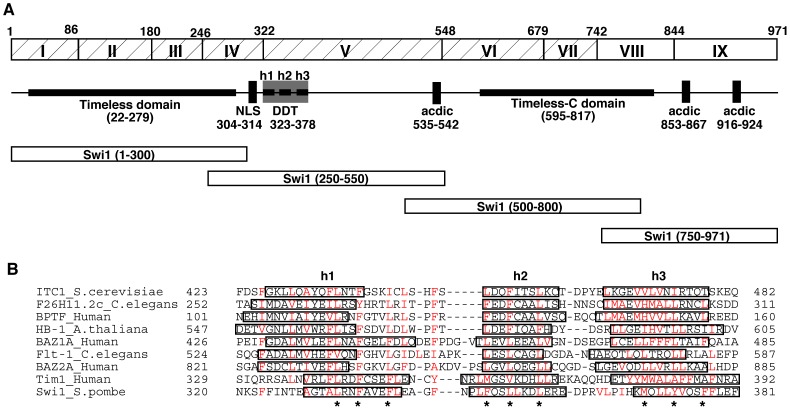
The structure of the *S. pombe* Swi1 protein. (A) The Swi1 polypeptide was divided into 9 putative functional sub-domains. Hatched boxes indicate the regions with amino acid sequences that are conserved throughout evolution. Swi1 contains the Timeless domain (22–279 aa), NLS (304–314 aa), the DDT domain (323–378 aa) and the Timeless-C domain (595–817 aa). h1, h2 and h3 in the DDT domain indicate alpha-helix regions. Swi1 also has stretches of acidic amino acids at 535–542, 858–867, and 916–924 aa regions. The four truncated versions of Swi1 constructed in this study are shown. aa, amino acid. (B) Multiple sequence alignment of DDT domains of various transcription factors, chromatin-remodeling proteins, human Tim1 (Timeless) and *S. pombe* Swi1. Conserved aromatic and hydrophobic residues are shown in red. The predicted helices are boxed. Asterisks indicate mutated amino acids in *swi1* mutants constructed in this study.

To understand the molecular basis of the Swi1-Swi3 complex, we conducted structural analyses of the Swi1 protein at the amino acid sequence level. We performed ClustalW multiple sequence alignment of Timeless-related proteins, including human Timeless, *Drosophila* Timeout, *C. elegans* Tim-1, *S. pombe* Swi1 and *S. cerevisiae* Tof1. This analysis predicted that Tof1 and Tim-1 have stretches of amino acid sequences that may divide Timeless-related proteins into at least 9 functional domains (Figure 1A and Figure S1). The three N-terminal domains have significant homology among the species and comprise the Timeless domain, which is the signature of Timeless-related proteins, as reported in the National Center for Biotechnology Information (NCBI) website (Figure 1A). The fourth domain also has significant similarity among the Timeless-related proteins and a putative nuclear localization signal (NLS) at amino acid 304–314 (Figure 1A). Interestingly, in the fifth functional domain, which is also conserved throughout evolution, we found the DDT (DNA-binding homeobox-containing proteins and the different transcription and chromatin remodeling factors in which it is found) domain (Figure 1A), which is a putative DNA binding domain found in various chromatin-remodeling factors [32]. This was revealed by computational protein motif searches using the Scansite program and the Jpred secondary structure prediction provided by the Massachusetts Institute of Technology and University of Dundee, respectively. The Scansite program detected a DDT domain in human Timeless, but not in *S. pombe* Swi1. However, the region containing the DDT domain was conserved between Timeless and Swi1 (Figure 1 and Figure S1). Therefore, we utilized Jpred secondary structure prediction to determine whether Swi1 contains a DNA binding consensus. This analysis found that Swi1 also has a DDT domain at amino acids 323–378 (Figure 1B). Similar to DDT domains from various chromatin-remodeling factors, the Swi1 DDT domain also consists of three alpha helices, each of which contains conserved aromatic or hydrophobic residues (Figure 1B). The sixth and seventh domains, which are part of the Timeless C-terminal domain (reported on NCBI website), also show similarity among species (Figure 1A). We were not able to detect significant conservation in the eighth and ninth domains. However, we found that the ninth domain of many species contains acidic amino acid stretches, which may have important functions (Figure 1A), although this is not the focus of the present study.

### Swi1 Facilitates the Recruitment of Swi3 to Chromatin

**Figure 2:**
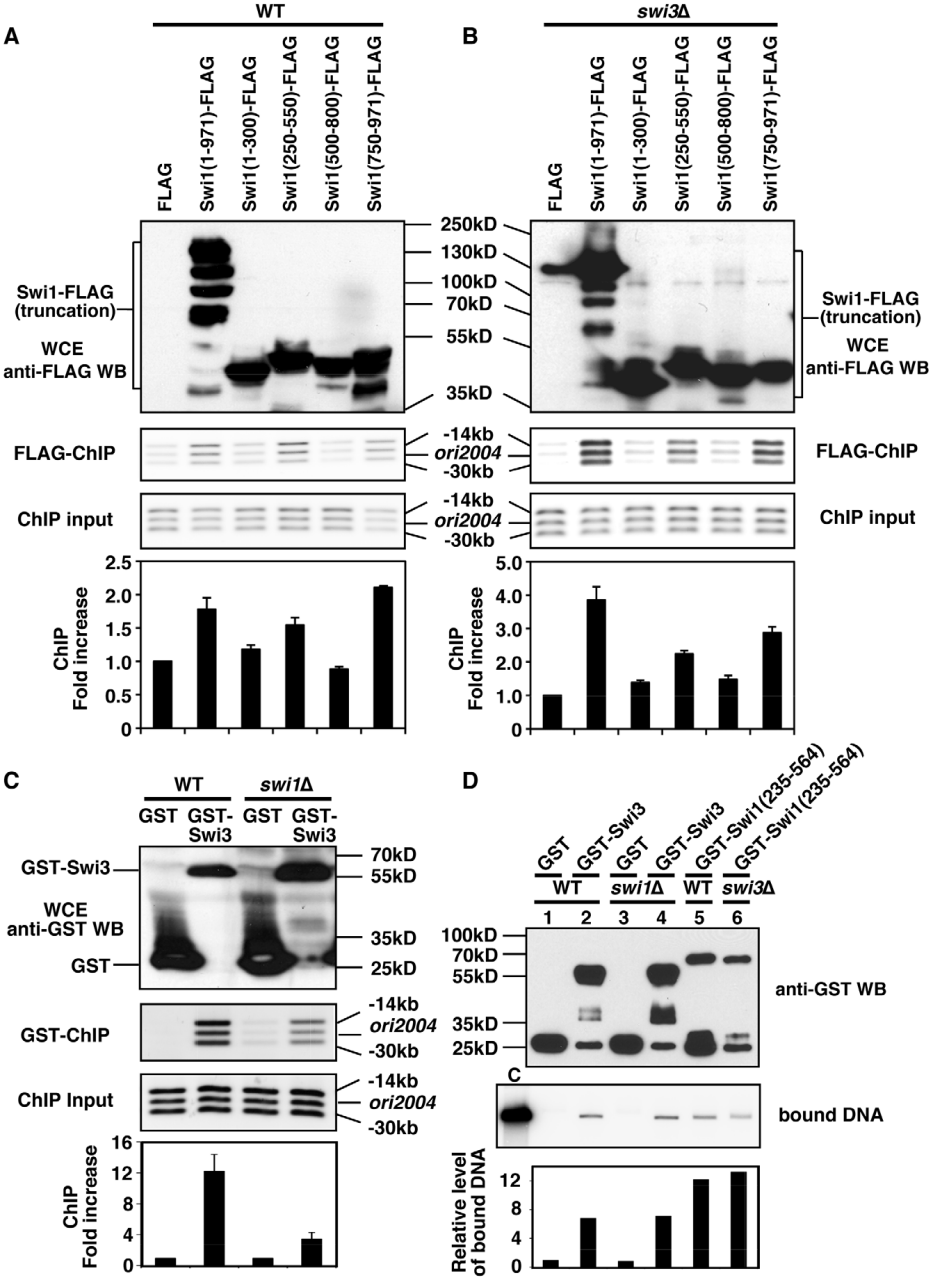
Swi1 facilitates the recruitment of Swi3 to chromatin. (A, B) ChIP assays of the indicated Swi1 truncated mutants were performed. Swi1 full-length (1–971), Swi1 (250–550), and Swi1 (250–550) precipitate the *ori2004* regions (*ori2004* and two positions 14- and 30-kb away from *ori2004*) in wild-type (WT, in A, middle panels) and *swi3*Δ mutants (in B, middle panels). Western blotting with anti-FLAG antibodies shows that all FLAG-fused Swi1 truncations mutants were similarly expressed (top panels). Fold increase in chromatin association over background (FLAG, set to 1) was calculated for each band. The average fold increase in the association of Swi1 truncations with the three positions (*ori200*4, −14 kb, and −30 kb) is shown, and error bars represent standard deviations obtained from the three positions (bottom panels). Representative results of repeat experiments are shown. WCE, whole cell extract; WB, Western blotting. (C) ChIP assay of GST-Swi3 was performed in wild-type or *swi1*Δ cells expressing the indicated proteins. GST-Swi3 strongly associates with the *ori2004* region in wild-type (WT), while GST-Swi3 has weak chromatin association in the absence of Swi1. Western blotting with anti-GST antibodies shows that GST-Swi3 was expressed similarly in wild-type and *swi1*Δ cells. Quantification of PCR bands was performed as described above. Fold increase in chromatin association over background (GST, set to 1) in each cell line (WT or *swi1*Δ) is shown. Representative results of repeat experiments are shown. (D) In vitro DNA binding assays of Swi1 (235–564) and Swi3 purified from the indicated *S. pombe* cells (top panel). The indicated proteins were mixed with radiolabeled plasmid pUC28, and associated DNA was analyzed by agarose gel electrophoresis (middle panel). Quantification of bound DNA was performed as described in Materials and Method. The values of bound DNA were normalized to the amount of proteins used in the reactions, and relative levels of bound DNA over background (GST) are shown. Representative results of repeat experiments are shown. C, input radiolabeled DNA.

Based on the aforementioned structural prediction, we generated a series of Swi1 truncation mutants (Figure 1A). These truncation mutants include the amino acid region 1–300 that comprises the Timeless domain, the amino acid region 250–550 that contains an NLS and the DDT domain, the amino acid region 500–800 that includes the Timeless C-terminal domain, and the amino acid region 750–971 that contains acidic amino acid stretches (Figure 1A). These regions were fused to 5FLAG at their C-termini and expressed under the control of the *nmt1* promoter in the wild-type *S. pombe* cells. As shown in Figure 2A, all truncation mutants were expressed at similar levels. To determine which region of Swi1 is required for its association with chromatin, chromatin immunoprecipitation (ChIP) was performed using anti-FLAG antibody (Figure 2A). We have previously demonstrated that Swi1-Swi3 associates with the *ori2004* region that contains an active replication origin [13], [33]. Therefore, Swi1-chromatin association was monitored at *ori2004* and two positions located 14- and 30-kb away from this origin. As expected, we observed that full length Swi1 (1–971) associates with the *ori2004* region (Figure 2A). In addition, we observed significantly stronger association of Swi1 (250–550) and Swi1 (750–971) with chromatin when compared to the background level (FLAG) (Figure 2A). Interestingly, this association was independent of Swi3, since full-length Swi1 (1–971), Swi1 (250–550) and Swi1 (750–971) were found to be associated with chromatin in *swi3*Δ cells (Figure 2B), suggesting that Swi1 may recruit Swi3 to chromatin. To address this question, we overexpressed GST-Swi3 in WT and *swi1*Δ cells and performed ChIP analysis using Glutathione Sepharose (Figure 2C). As shown in Figure 2C, strong chromatin association of GST-Swi3 was detected in wild-type cells. However, this association was significantly reduced in the absence of Swi1 (Figure 2C). Thus, these results indicate that although Swi3 has the ability to interact with chromatin in a manner independent of Swi1, Swi1 is required for the efficient association of Swi3 with chromatin.

### The DTT Domain-containing Region of Swi1 and Swi3 Interact with DNA in vitro

ChIP experiments described above identified the 250–550 amino acid region as a domain involved in the association of Swi1 with chromatin (Figure 2). Interestingly, we found that this region contains the DDT domain (amino acids 323–378, Figures 1A and 1B), a putative DNA binding domain. To further understand the mechanisms by which Swi1-Swi3 interacts with chromatin, we determined whether the DDT domain-containing region directly interacts with DNA in vitro. For this purpose, the amino acid region 235–564 of Swi1 was fused to GST and expressed under the control of the *nmt1* promoter in *S. pombe* cells. Swi1 (235–564) was then purified using Glutathione Sepharose under a stringent condition as described in Materials and Methods. This approach was chosen due to technical difficulties in purifying the Swi1 truncation mutant using *E. coli*. To determine whether Swi1 (235–564) binds DNA, GST-Swi1 (235–564) was incubated with a ^32^P-labelled plasmid DNA. Consistent with our ChIP results (Figures 2A and 2B), this investigation revealed that Swi1 (235–564) was able to bind the plasmid DNA, whereas GST alone failed to do so (Figure 2D, lanes 1 and 5). In addition, Swi1 (235–564) purified from *swi3*Δ cells bound DNA (Figure 2D, lane 6). Furthermore, we found that GST-Swi3 purified from both wild-type and *swi1*Δ cells binds DNA (Figure 2D, lanes 1–4). These results suggest that both the DDT domain-containing region of Swi1 and Swi3 directly bind DNA.

### The DDT Domain of Swi1 is Involved in the Recruitment of Swi1-Swi3 to Chromatin

**Figure 3:**
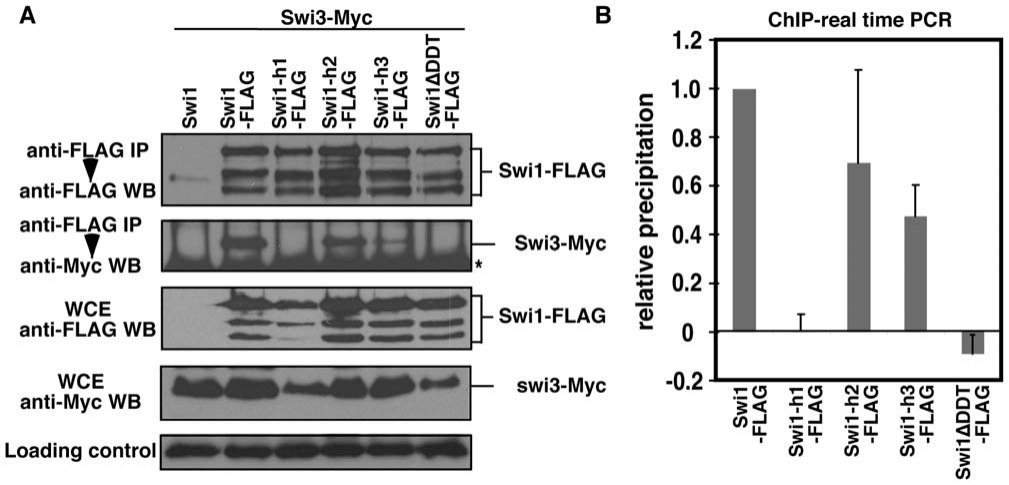
Swi1 associates with chromatin through the DDT domain. (A) Mutations in the DDT domain abolished Swi1-Swi3 complex formation. Protein extracts were prepared from cells expressing the indicated fusion proteins. Swi1-FLAG was immunoprecipitated and probed with the anti-FLAG M2 antibody (top panel) or the anti-Myc 9E10 antibody (second panel). Swi1-FLAG was equally precipitated in all of the mutants while Swi3-Myc failed to co-purify with Swi1-FLAG in *swi1-h1* and *swi1*Δ*DDT* mutants. Swi1-FLAG is shown to be similarly expressed in all *swi1* mutants (third panel), while the expression levels of Swi3-Myc were decreased in *swi1-h1* and *swi1* ^Δ*DDT*^ mutants (fourth panel). The appearance of three bands in Swi1-Myc Western blots is due to degradation of the fusion protein [13], [14], [31]. Asterisk shows non-specific bands. Representative results of repeat experiments are shown. (B) ChIP assays of Swi1-FLAG were performed on cell extracts prepared from the indicated strains. Association of Swi1-FLAG mutants with chromatin was monitored at *ori2004*. Swi1-h1 and Swi1^ΔDDT^ failed to associate with chromatin. As described in Materials and Methods, the relative precipitation value of wild-type Swi1-FLAG was set to 1. Error bars correspond to standard deviations obtained from at least three independent experiments.

The DNA-binding ability of the DDT domain-containing region of Swi1 suggests the possibility that Swi1 interacts with chromatin through its DDT domain. Therefore, we mutated the conserved aromatic or hydrophobic residues in each alpha helix of the DDT domain (*swi1-h1* for *swi1^L333A, F336A, F340A^-5FLAG*; *swi1-h2* for *swi1^F349A, L352A, L356A^-5FLAG*, and *swi1-h3* for *swi1^Y373A, F377A, F378A^-5FLAG*, Figure 1B). We also generated a *swi1* mutant that contains a deletion of the DDT domain (amino acids 323–378; *swi1* ^Δ*DDT*^ for *swi1* ^Δ*DDT*^ *-5FLAG*). These mutants were integrated at the *leu1* locus of a *swi1*Δ *swi3-Myc* strain and expressed from the *swi1* promoter. As shown in Figure 3A, all Swi1-FLAG mutants were similarly expressed in and immunoprecipitated from *S. pombe* cells. To investigate whether the DDT domain is required for the association of Swi1 with chromatin, we performed ChIP analyses of these Swi1 mutants using quantitative real-time PCR. Accordingly, we found that Swi1-h1 and Swi1^ΔDDT^ lost chromatin association whereas Swi1-h2 and Swi1-h3 retained chromatin interaction. However, the levels of Swi1-chromatin association were somewhat reduced in *swi1-h2* cells and significantly low in *swi1-h3* cells (Figure 3B). These data indicate that the first alpha helix of the DDT domain has a major role in the association of Swi1 with chromatin. The results also suggest that the second and the third helices may have a minor role in Swi1-chromatin interaction.

We also examined whether Swi3 is able to associate with Swi1 in the DDT domain mutants. Interestingly, we found by immunoprecipitation, that Swi1-h1 and Swi1^ΔDDT^ lost interaction with Swi3, whereas Swi1-h2 was able to associate with Swi3 in cell extracts (Figure 3A). In addition, Swi1-Swi3 interaction was considerably compromised in *swi1-h3* cells. Furthermore, Swi3 protein levels were significantly reduced in *swi1-h1* and *swi1* ^Δ*DDT*^ mutants (Figure 3A), suggesting that the association of Swi1 with chromatin is involved in Swi1-Swi3 complex formation and stability of these proteins. Therefore, our data suggest that the first alpha helix of the DDT domain plays a critical role in the formation of the Swi1-Swi3 complex and the stability of Swi3 in *S. pombe* cells. It is also possible that the third helix is involved in Swi1-Swi3 complex formation.

### Domains of Swi1 Required for Swi1-Swi3 Complex Formation

**Figure 4:**
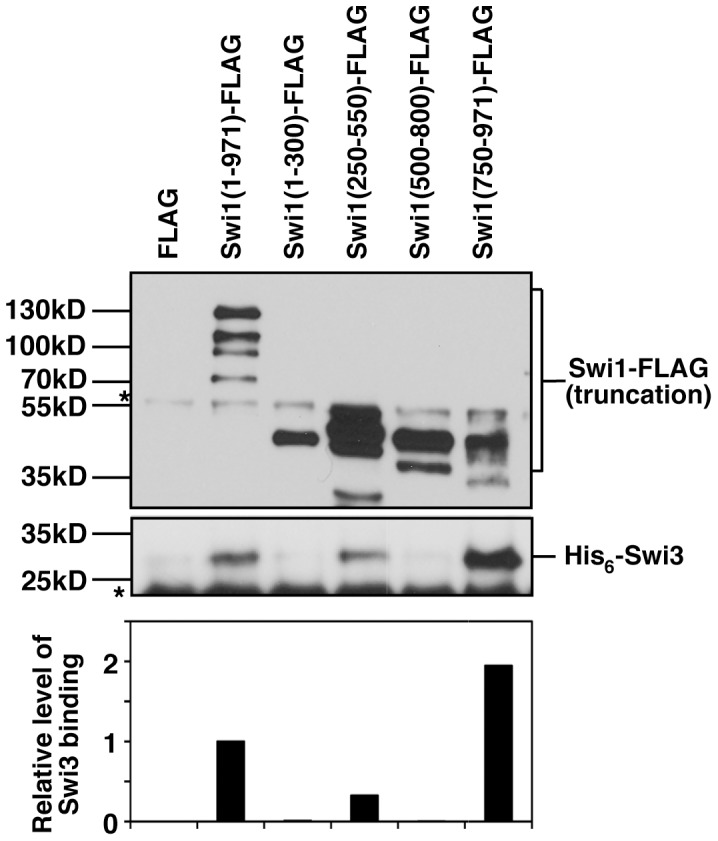
Domains of Swi1 required for Swi1-Swi3 complex formation. The indicated Swi1 truncations mutants fused to FLAG were expressed in and purified from *swi3*Δ cells (top panel). Anti-FLAG agarose beads bound to the indicated Swi1 truncation mutant were incubated with recombinant His_6_-Swi3. The beads were washed and analyzed by Western blotting using the anti-FLAG or His_6_ antibody (middle panel). Asterisks indicate non-specific bands. Quantification of His_6_-Swi3 bands was performed using EZQuant, normalizing the values to the amounts of Swi1 truncations used in the reactions. Swi3 binding activity of Swi1 (1–971) was set to 1. Representative image of repeat experiments are shown.

Our results suggest that the DDT domain-containing region (Swi1 250–550 amino acids) is required for Swi1-Swi3 complex formation. To further test this possibility, various truncated versions of Swi1 (1–300, 250–550, 500–800, 750–971 amino acid regions) fused to 5FLAG at the C-terminus were again expressed under the control of the *nmt1* promoter. To characterize the properties of Swi1 truncation mutants in the absence of Swi3, these FLAG-tagged proteins were purified from *swi3*Δ cells using anti-FLAG antibody under a stringent condition as described in Materials and Methods. This approach was again chosen due to technical difficulties in purifying Swi1 truncation mutants using *E. coli*. To determine the regions of Swi1 that interact with Swi3, hexahistidine-tagged Swi3 (His_6_-Swi3) was expressed in and purified from *E. coli*. Accordingly, recombinant His_6_-Swi3 was tested for its ability to interact with the 5FLAG-tagged Swi1 truncation mutants that were purified from *swi3*Δ cells. Previous studies in human cells have reported that the overexpressed N-terminal 1–573 amino acid region of Timeless (Swi1 homolog) is responsible for interacting with overexpressed Tipin (Swi3 homolog) [28]. Consistently, our in vitro analysis revealed that full-length Swi1 (1–971) and Swi1 (250–550) interact with His_6_-Swi3, although Swi1 (1–300) and Swi1 (500–800) failed to associate with Swi3 (Figure 4). In addition, we found that C-terminal Swi1 (750–971) was also able to bind His_6_-Swi3 (Figure 4). Interestingly, Swi1 (750–971) had stronger Swi3-binding activity when compared to that of Swi1 (1–971) or Swi1 (250–550). This result suggests that Swi1 (750–971) has stronger affinity with Swi3 than Swi1 (250–550) and that Swi1 contains domains that hinder Swi1-Swi3 interaction. Taken together, our results suggest that multiple domains in Swi1 are involved in Swi1-Swi3 complex formation.

### The DDT Domain is Involved in the Functions of Swi1

**Figure 5:**
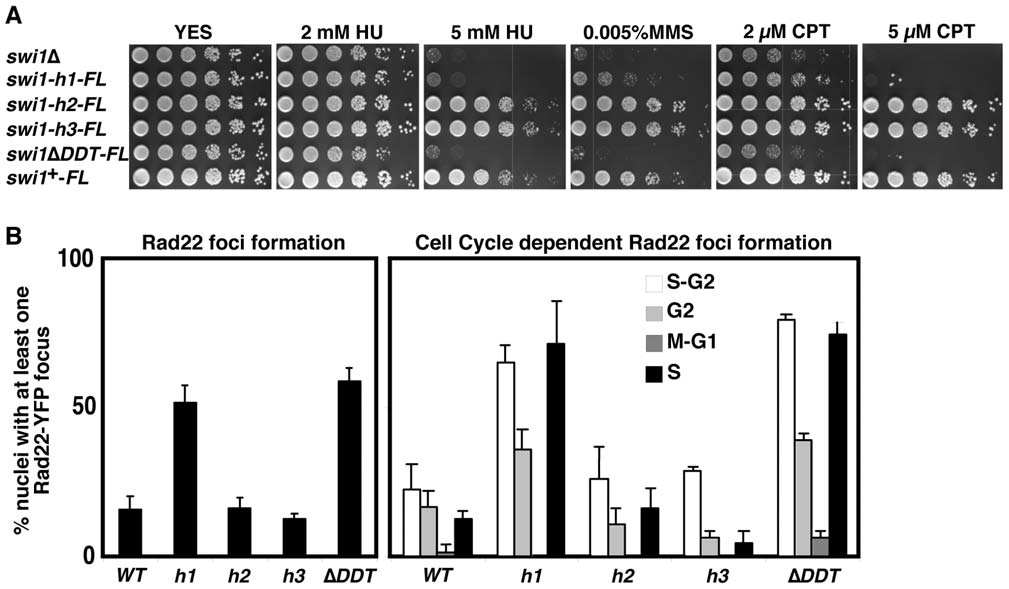
DDT domain is essential for Swi1’s functions. (A) Five-fold serial dilutions of the indicated cells were incubated on YES agar medium supplemented with the indicated drugs for 2 to 4 days at 32°C. Representative images of repeat experiments are shown. (B) Cells of indicated *swi1* mutants were engineered to express Rad22-YFP and grown in YES medium at 25°C until midlog phase. The percentages of nuclei with at least one Rad22-YFP focus are shown (left panel). At least 200 cells were counted for each strain. Error bars correspond to standard deviations obtained from at least three independent experiments. Quantification of Rad22-YFP foci according to the cell cycle stages was also performed by analyzing cell length, nuclei number and position, and the presence of a division plate, as described in our previous publications (right panel) [13], [15], [19], [43], [55]. Schematic drawing for nuclear and morphological changes during the *S. pombe* cell cycle is shown (right panel). *swi1-h1* and *swi1* ^Δ*DDT*^ cells displayed strong accumulation of Rad22-YFP foci during S and early G2 phases.

Our results suggest that the DDT domain is involved in Swi1’s functions. *swi1* deletion has been reported to render cells highly sensitive to various genotoxic agents, including hydroxyurea (HU), methyl methanesulfonate (MMS), and camptothecin (CPT), all of which are known to affect S-phase progression. As shown in Figure 5A, *swi1-h1* and *swi1* ^Δ*DDT*^ cells, which have defects in the association of Swi1 with chromatin, showed hypersensitivities to these genotoxic agents, as was the case for *swi1*Δ cells. In contrast, *swi1-h2* and *swi1-h3*, which retain Swi1-chromatin interaction, showed no significant sensitivity to genotoxic agents.

We previously reported that *swi1* deletion results in an accumulation of DNA damage due to replication problems. Therefore, to examine the functionality of Swi1 mutants, we also monitored Rad22-YFP DNA foci formation in the absence of genotoxic agents. *swi1-h1* and *swi1* ^Δ*DDT*^ cells displayed strong accumulation of Rad22-YFP foci during S phase, whereas *swi1-h2* and *swi1-h3* showed Rad22-YFP foci formation similar to wild-type cells (Figures 5B). Taken together, our results are consistent with the notion that Swi1 associates with chromatin through its DDT domain and recruits Swi3 to chromatin in order to function as the replication fork protection complex and preserve genomic integrity.

## Discussion

In a previous study, the Swi1-Swi3 replication fork protection complex was purified from *E. coli* cells co-expressing Swi1 and Swi3. This preparation of the Swi1-Swi3 complex directly interacted with duplex DNA without structural preference [17]. It has also been reported that Swi1-Swi3 facilitates DNA binding of Mrc1 to promote replication checkpoint response [16], [34]. However, how Swi1 and Swi3 form a complex and associate with chromatin is poorly understood. To understand the molecular basis of the Swi1-Swi3 complex, we performed domain analysis of Swi1 in fission yeast. Importantly, our studies revealed that Swi1 contains the DDT domain, which is essential for Swi1’s ability to interact with chromatin. The DDT domain contains about 60 amino acids with three alpha helices, and similar alpha helical composition is often found in various DNA binding domains of known structure [32]. Our investigation demonstrated that these alpha helices are important for Swi1’s chromatin association. Mutations in the first alpha helix caused the complete loss of chromatin association of Swi1, while the third alpha helix mutations led to some reduction of chromatin association (Figure 3B). In addition, we found that the DDT domain-containing region of Swi1 (235–564 amino acids) is able to interact with DNA in vitro (Figure 2D). Considering that the DDT domain is a putative DNA-binding domain found in various chromatin remodeling factors [32], our results suggest that Swi1 interacts with DNA via the DDT domain.

Interestingly, mutations in the DDT domain also abolished Swi1-Swi3 complex formation in the immunoprecipitation experiments using cell extracts. In addition, the DDT domain-containing region (amino acids 250–550) purified from *S. pombe* cells also interacted with recombinant Swi3. This is consistent with the fact that the 267–573 amino acid region of Timeless is able to interact strongly with Tipin in immunoprecipitation experiments using human cell extracts [28]. Although we purified Swi1 truncation mutants using a stringent condition to minimize co-purification of associated factors, we cannot exclude the possibility that Swi1 (250–550) interacts with other cellular proteins such as replisome components, which could mediate Swi1-Swi3 interaction in cell extracts.

It appears that Swi1-Swi3 interaction is not simply mediated by a single domain of Swi1. Interestingly, we found that the Swi1 C-terminal region (750–971) also interacts with Swi3. Furthermore, it has been reported that F660A/I661I, Y26A, Y42A, Y127A/K128A, or R268A/H269A mutations in Swi1 also destabilize Swi1-Swi3 complex formation [17]. It is also possible that these point mutations cause structural defects of Swi1 leading to disruption of the Swi1-Swi3 complex [17]. However, it is likely that there are multiple potential Swi3 binding sites within the Swi1 polypeptide and that these binding interfaces cooperate together to promote tight Swi1-Swi3 complex formation, which is required for tolerance to S-phase stressing agents.

Interestingly, Swi1-Swi3 complex formation was also found to be important for the stability of Swi3. *swi1-h1* and *swi1* ^Δ*DDT*^ mutant cells, in which Swi1-Swi3 complex formation is lost, had lower levels of Swi3 (Figure 3A). Our previous results also demonstrated that Swi3 mutant cells that abolish Swi1-Swi3 complex formation display reduced levels of Swi3 [31]. Since Swi1 is required for the efficient association of Swi3 with chromatin (Figure 2C), our data may suggest that Swi3 becomes unstable when it is dissociated from chromatin, implying that Swi1 forms a complex with Swi3 on chromatin. It is possible that Swi1 directly binds DNA through the DDT domain and recruits Swi3 to chromatin and that Swi3 is degraded once it leaves chromatin. However, our in vitro experiments demonstrated that Swi1 interacts with Swi3 in the absence of DNA (Figure 4). Therefore, it is also possible that Swi1 and Swi3 form a complex before they interacts with chromatin. Nevertheless, our study demonstrated that Swi1 enhances the ability of Swi3 to interact with chromatin (Figure 2C). In addition, Swi3 overexpression does not suppress defects associated with *swi1* deletion [13], further supporting our conclusion that Swi1 is required for Swi3’s efficient association with chromatin. It is also possible that Swi1 associates with chromatin via replisome components during DNA replication, as previous studies have reported that Swi1 or its homologs interact with the MCM complex and Cdc45 [25], [35], [36]. Therefore, it is important for future studies to investigate whether Swi1 directly associates with DNA at replication forks and how Swi1 is recruited to the replication fork.

Our studies have also revealed that the DDT domain is essential for Swi1’s functions in DNA damage tolerance and prevention of DNA damage (Figure 5). Currently it remains unclear whether the loss of Swi1-chromaitin association or loss of Swi1-Swi3 complex formation primarily causes defects in DNA damage tolerance. This is due to the lack of Swi1 mutants only affecting Swi1’s DNA-binding ability or Swi3-binding ability. However our results suggest that the DDT domain has an essential role in Swi1’s functions as a subunit of the fork protection complex. Our results also indicate that Swi1-Swi3 complex formation plays a critical role in activation of the replication checkpoint and preservation of stable replication fork structures in response to exposure to genotoxic agents. Swi1-Swi3 is also required for unperturbed DNA replication, as shown by the strong accumulation of Rad22-YFP foci during S-phase in *swi1* DDT mutants, in the absence of genotoxic agents (Figure 5). An increasing number of chromatin-associated proteins are reported to contain the DDT domain [37], [38]. Therefore, we suspect that Swi1-Swi3 may be involved in coordinating chromatin organization at the replication forks. Interestingly, large-scale synthetic lethal analyses found that *swi3* is synthetically lethal with mutations in various chromatin-remodeling factors [39], [40]. These findings suggest that proper chromatin remodeling requires intact replication fork structures, which are secured by the Swi1-Swi3 complex. Further investigation is required to explore this possibility.

## Materials and Methods

### General Techniques

The methods used for genetic and biochemical analyses of fission yeast have been described previously [41], [42]. PCR amplification of DNA was done using EX taq DNA polymerase (TaKaRa, Ohtsu, Japan). Accurate PCR reactions were confirmed by DNA sequencing analyses. Microscopic analyses of yellow fluorescent protein (YFP), Western blotting, and drug sensitivity assays were performed as described in our earlier studies [13], [15], [31], [43], [44]. For protein overexpression from the thiamine-repressive *nmt1* promoter, *S. pombe* cells were first grown in the presence of thiamine until mid-log phase, then washed three times with culture medium without thiamine, grown again for 18 hours, and collected for protein purification or ChIP. For immunoblotting, Myc, GST, and FLAG fusion proteins were probed with the anti-c-Myc 9E10 monoclonal antibody (Covance, Berkeley, CA), the anti-GST antibody, and the anti-FLAG M2 monoclonal antibody (Sigma-Aldrich), respectively.

### Plasmids

The plasmids used in this study are listed in Table 1.

Plasmids used for overexpression of Swi1 and Swi3: The 0.4 kb BamHI-BglII fragment containing five tandem copies of the *FLAG* sequence (5FLAG) and the sequence of *S. cerevisiae ADH1* terminator was excised from pFA6a-5FLAG-kanMX6 [45] and introduced into the BamHI site of pREP1 [46], resulting in pREP1-5FLAG. To express C-terminal 5FLAG-tagged full length Swi1 (1–971 amino acids) and truncated versions (1–300, 250–550, 500–800 and 750–971 amino acids regions) in *S. pombe* cells, each corresponding *swi1* coding region was amplified by PCR and inserted into the SalI/BamHI site of pREP1-5FLAG. The amino acid region 235–564 of *swi1* and the *swi3* open reading frame were amplified from *S. pombe* genomic DNA by PCR and fused to GST in pREP-KZ [47], resulting in pREP-GST-Swi1 (235–564) and pREP-GST-Swi3, respectively. For bacterial expression of hexahistidine-tagged Swi3 (His_6_-Swi3), the *swi3* open reading frame was inserted into the NdeI/XhoI site of pET28a (Novagen), resulting in pET28a-Swi3.

**Table 1:** Plasmids used in this study.

Plasmid	Relevant genes	Source
pREP-KZ	*Pnmt1* * *-GST*, *LEU2*	[56]
pREP-GST-Swi1(235–564)	*Pnmt1-GST-Swi1*(235–564), *LEU2*	This study
pREP-GST-Swi3	*Pnmt1*-*GST-Swi3*, *LEU2*	This study
pREP-5FLAG	*Pnmt1-5FLAG*, *LEU2*	This study
pREP-Swi1-5FLAG	*Pnmt1*-*5FLAG-Swi1*, *LEU2*	This study
pREP-Swi1(1–300)-5FLAG	*Pnmt1*-*5FLAG-Swi1*(1–300), *LEU2*	This study
pREP-Swi1(250–550)-5FLAG	*Pnmt1*-*5FLAG-Swi1*(250–550), *LEU2*	This study
pREP-Swi1(500–800)-5FLAG	*Pnmt1*-*5FLAG-Swi1*(500–800), *LEU2*	This study
pREP-Swi1(750–971)-5FLAG	*Pnmt1*-*5FLAG-Swi1*(750–971), *LEU2*	This study
pJK148-swi1-5FLAG	*swi1-5FLAG*, *leu1* ^+^	This study
pJK210-rad22CT-YFP	*rad22CT-YFP*, *ura4* ^+^	This study
pET28a-Swi3	*His_6_-Swi3*, *amp* ^r^	This study

* The *S. pombe nmt1* promoter for overpexpression of GST- or FLAG-fused proteins.

Plasmid used to observe Rad22-YFP foci: The 1.5 kb NotI-BglII fragment containing a C-terminal *rad22* region fused with *YFP* cDNA (*rad22CT-YFP*) [15], [48] was introduced into the NotI/BamHI site of pJK210 [49], resulting in pJK210-rad22CT-YFP.

Plasmids used for site-directed mutagenesis: The 3.3 kb *swi1* genomic fragment including the *swi1* promoter region was amplified by PCR from wild-type *S. pombe* genomic DNA to eliminate the *swi1* stop codon and fused to the PCR amplified 5FLAG sequence in pJK148, resulting in pJK148-swi1-5FLAG.

### *S. pombe* Strains

The *S. pombe* strains used in this study were constructed using standard techniques [41], and their genotypes are listed in Table 2. *swi3-13Myc* (*swi3-13Myc*:*hphMX6*) was generated by a one-step marker switch method [50] using the *swi3-13Myc*:*kanMX6* strain. *swi1* DDT domain mutants were generated by Kunkel site-directed mutagenesis [51] in pJK148-swi1-5FLAG and integrated at the *leu1* locus of a *swi1*::*kanMX6 swi3-13Myc*-*hphMX6* strain. To visualize Rad22-YFP in *swi1* mutants, pJK210-Rad22CT-YFP was integrated at the *rad22* locus of *swi1* mutant strains.

**Table 2:** *S. pombe* strains used in this study.

Strain	Genotype*	Source
Y0001	*h* ^−^	[19]
Y0211	*h* ^−^ *swi1*::*kanMX6*	[19]
Y0668	*h* ^−^ *swi3*::*kanMX6*	[19]
Y2467	*h* ^+^ *swi1*::*kanMX6 swi3-13myc*:*hphMX6*	This study
Y2589	*h* ^+^ *swi1*::*kanMX6 swi3-13myc*:*hphMX6 leu1* ^+^:*swi1-5FLAG*	This study
Y2590	*h* ^+^ *swi1*::*kanMX6 swi3-13myc*:*hphMX6 leu1* ^+^:*swi1-h1-5FLAG*	This study
Y2591	*h* ^+^ *swi1*::*kanMX6 swi3-13myc*:*hphMX6 leu1* ^+^:*swi1-h2-5FLAG*	This study
Y2592	*h* ^+^ *swi1*::*kanMX6 swi3-13myc*:*hphMX6 leu1* ^+^:*swi1-h3-5FLAG*	This study
Y2593	*h* ^+^ *swi1*::*kanMX6 swi3-13myc*:*hphMX6 leu1* ^+^:*swi1*Δ*DDT-5FLAG*	This study
Y2705	*h* ^+^ *swi1*::*kanMX6 swi3-13myc*:*hphMX6 leu1* ^+^:*swi1-5FLAG rad22-YFP*:*ura4* ^+^	This study
Y2706	*h* ^+^ *swi1*::*kanMX6 swi3-13myc*:*hphMX6 leu1* ^+^:*swi1-h1-5FLAG rad22-YFP*:*ura4* ^+^	This study
Y2708	*h* ^+^ *swi1*::*kanMX6 swi3-13myc*:*hphMX6 leu1* ^+^:*swi1-h2-5FLAG rad22-YFP*:*ura4* ^+^	This study
Y2710	*h* ^+^ *swi1*::*kanMX6 swi3-13myc*:*hphMX6 leu1* ^+^:*swi1-h3-5FLAG rad22-YFP*:*ura4* ^+^	This study
Y2712	*h* ^+^ *swi1*::*kanMX6 swi3-13myc*:*hphMX6 leu1* ^+^:*swi1* ^Δ*DDT*^ *-5FLAG rad22-YFP*:*ura4* ^+^	This study

* All strains are *leu1-32* and *ura4-D18*.

Mutations and epitope-tagged genes have previously been described for *swi1*Δ (*swi1*::*kanMX6*) [15]; *swi3*Δ (*swi3*::*kanMX6*), and *swi3-13Myc* (*swi3-13Myc*:*kanMX6*) [13].

### Chromatin Immunoprecipitation (ChIP) Assay

ChIP assay was performed essentially as described in our earlier studies [13], [19], [44]. Briefly, *S. pombe* cells (5×10^8^) were fixed in 1% formaldehyde for 20 min at room temperature and quenched in 125 mM glycine for 5 min. Cells were then washed in TBS and disrupted in lysis buffer (50 mM Hepes-KOH pH7.5, 140 mM NaCl, 1 mM EDTA, and 1% Triton X-100) supplemented with protease inhibitors {0.2 mM p-(Amidinophenyl) methanesulfonyl fluoride (*p*-APMSF) and Roche protease inhibitor cocktail}. The broken cells were sonicated 12 times for 20 seconds each with a Misonix Sonicator 3000 until chromatin DNA was sheared into 500 to 700 bp fragments. The cell lysate was clarified by two rounds of maximum speed centrifugation in an Eppendorf 5415C microcentrifuge at 4°C. Immunoprecipitations were performed in these cell extracts using anti-FLAG M2 agarose or Glutathione Sepharose 4B beads. DNA recovered from these beads was analyzed by regular PCR or triplicate SYBR Green-based real-time PCR (Bio-Rad). Amplification conditions and the specific primers used in regular PCR have been described previously [33]. Quantification of regular PCR bands was performed using NIH ImageJ, normalizing the values to the amount of input DNA bands. The primers, conditions and the calculation method used in real-time PCR ChIP analysis were described in our previous publication [52]. Briefly, raw percent precipitated DNA values (percentage raw-precipitation) were calculated based on ΔCt between input DNA and immunoprecipitated DNA. ChIP analyses were also performed using strains expressing untagged proteins to obtain percentage background-precipitation values, and they were subtracted from percentage raw-precipitations values to obtain percentage precipitation values. To compare ChIP data between different *swi1* mutants, we converted the percentage precipitation values to relative precipitation values by setting the percentage precipitation values from wild-type Swi1-FLAG experiments to 1.

### In vitro DNA-binding Assay

In vitro DNA-binding assay was performed as described previously with minor modifications [53], [54]. The pUC28 plasmid DNA was digested by EcoRI and 5′ end-labeled using the T4 polynucleotide kinase and [γ^32^P]-ATP. Sepharose beads bound to GST fusion proteins were equilibrated with binding buffer B (10 mM Tris-Cl pH 7.5, 1 mM EDTA, 0.5% NP-40), mixed with the radiolabeled pUC28 plasmid DNA, and incubated by rotation at 37°C for 10 min. After incubation, the beads were spun down, washed twice with binding buffer B, and incubated with 2 µg/µl proteinase K at 37°C for 15 min. The beads were spun down again, and the supernatant containing DNA was analyzed by 0.7% agarose electrophoresis in TAE buffer. The gel was dried and analyzed with a Molecular Dynamics Storm 840 phosphorimager (Molecular Dynamics, GE Healthcare). Radiolabeled DNA bands on the gel were quantified using ImageQuant (Molecular Dynamics, GE Healthcare), normalizing the values to the level of proteins used in DNA-binding reactions.

### Protein Purification and in vitro Protein Interaction Assay

For purification of FLAG-tagged proteins from *S. pombe* cells, cells expressing FLAG-tagged proteins were cultured in YES medium and collected when an optical density of 1.2 at 600 nm was reached. Cells were then lysed with glass beads in lysis buffer A {50 mM Tris-HCl (ph 8.0), 150 mM NaCl, 0.1% NP-40, 10% glycerol, 50 mM NaF, 1 mM Na_3_VO_4_, 5 mM EDTA, 5 mM *N*-methylmaleimide, 1 µM microcyctin, 0.1 µM okadaic acid, 0.2 mM *p*-4-amidoinophenyl-methane sulfonyl fluoride hydrochloride monohydrate (*p*-APMSF) and Roche complete EDTA-free protease inhibitor cocktail} using a FastPrep cell disrupter (Qbiogene) for two cycles of 20 seconds each at speed 6, with a one-minute interval on ice between the two cycles. Protein extracts were clarified by centrifugation at 13,000 rpm in an Eppendorf microcentrifuge 5415D for 10 min at 4°C, mixed with anti-FLAG M2 agarose (Sigma-Aldrich) and incubated for 2 hr at 4°C. The agarose beads were collected, washed three times in lysis buffer B (lysis buffer A with 500 mM NaCl), and stored in lysis buffer A.

Purification of GST-fused proteins from *S. pombe* cells was performed as described above except that Glutathione Sepharose 4B (GE Healthcare) was used in place of anti-FLAG M2 agarose.

*E. coli* BL21(DE3) cells expressing His_6_-Swi3 were suspended in lysis buffer H (50 mM NaH_2_PO_4_ pH 8.0, 300 mM NaCl, 10% Glycerol, 0.25% Tween 20, 10 mM β-mercaptoethanol, and 1 mM PMSF) containing 10 mM imidazole and lysed by sonication using a Branson Digital Sonifier. The lysate was clarified by centrifugation (Beckman JA-17 rotor, 15 krpm, 30 min, 4°C) and mixed with Ni-NTA (Qiagen) beads for 1 hour at 4°C. The Ni-NTA beads were washed in lysis buffer H containing 20 mM imidazole, and His_6_-Swi3 was eluted with lysis buffer H containing 250 mM imidazole and dialyzed against lysis buffer A.

Anti-FLAG M2 agarose beads bound to Swi1-FLAG were mixed with His_6_-Swi3 in lysis buffer A, incubated by rotation for 1 hour at 4°C, washed three times in lysis buffer A, and analyzed by Western blotting. Quantification of proteins bands was performed using EZQuant software (EZQuant).

## Supporting Information

Figure S1 **ClustalW multiple alignments of human Timeless,** ***Drosophila*** **Timeout,** ***C. elegans*** **Tim-1,** ***S. pombe*** **Swi1 and** ***S. cerevisiae*** **Tof1.**
(PDF)
