# Supplementary material for: Swi1 Associates with Chromatin through the DDT Domain and Recruits Swi3 to Preserve Genomic Integrity
Source: PLoS One. 2012 Aug 30;7(8):e43988. doi: 10.1371/journal.pone.0043988 (PMC3431386; doi:10.1371/journal.pone.0043988)
Supplement: Figure S1 — ClustalW multiple alignments of human Timeless, Drosophila Timeout, C. elegans Tim-1, S. pombe Swi1 and S. cerevisiae Tof1. (PDF) [file pone.0043988.s001.pdf]

Supplemental Figure S1

```
Timeless      -----------------MDLHMMNCELLATCSALGYLEGD---TYHKEPDCLESVKDLIR
Timeout       -------------------MSILLADIDATCAALGYSDGQ---KYQAEPDAAEGLKHLIW
Tim-1         ----------------------MNVLVQGAVHALGYYEDG---KYSREPDCYESIRDLIR
Swi1      1   -----------------MELDEVIQGIVSAIGGFDYSDDEK--VYVLGDEALACLKDLKR  41
Tof1          MSADLQQGTTNAADFSLTVLRARIALLATAIGGPDYTSQIDPPPYKLGDDCLACLKDLKR

Timeless      YLRHEDET---RDVRQQLGAAQILQSDLLPILTQHHQDKPLFD-----------------
Timeout       ILRRDLDN---HEYRRHLGRSKVLQTDLVYMLPDYVHHEELSD-----------------
Tim-1         YLREDGDD---HTARIECGRHNLVEQDLVPMVKCEDLTDDEFD-----------------
Swi1     42   YLQVVDEKYKVWQIRSLLSSLQLVTNDICPILSDWDKDITNYRNW---------------  86
Tof1          WFKLVDDQQKRWDVAMAVAEYRILTDDLLPILIDWENKCSLAAKLAKNNPDHEEFRNKAY

Timeless      --AVIRLMVNLTQPALLCFGN-LPKEPSFRHHFLQVLTYLQAYKEAFASEKAFGVLSETL
Timeout       --LLIRLLVILTNPTLLLYREGAPKDNHGRKVFMELIDILQGYKAAFAKDKVWSALFEKL
Tim-1         --IAIRLMVNLCQPAISTMRGKPPADRDQWKMYWELEENLRRAKTAFSDAHFFTAIKKRI
Swi1     87   --RIALACVELLVPLTWPLETEHETFRENVDVLYNLRQAQSNYKNSILSYKKGSVLSAIL  144
Tof1          YDKIALNCLQLLVLMTWPLIVTEQSSSNQITLYGELKKHQLVYKKTILSMESGKVLRAAI

Timeless      Y---ELLQLGWEERQEEDNLLIERILLLVRNILHVP------------------ADLDQE
Timeout       K---QALEIAFAIRSEEQNLLIERILVLVRNVLQVP------------------ANPEAE
Tim-1         DN--YFIDTEYEDRDERLRLVVERIVLLIKYVFSIN------------------PDTSEG
Swi1    145   AVLLKPLSTPAESRTLRDKGIIRIVLLLFRNILQID------------------ELKTKN  186
Tof1          RLALDVIKIDRLSRTPRDNMVLKLVLNFFRNVIAIEPGEFTINTKKSMPKKGITSIDTLP

Timeless      KKIDDDASAHDQLLWAIHLSGLDDLLLFLASSS----AEEQWSLHVLEIVSLMFRDQNPE
Timeout       CRADNDASLHDQVIWALHQTGMLDLVLFVISSP----DEEQFHLHGLEIVCLLFREQSAE
Tim-1         RRTRIEDSSHDRVIIAFLESGIDKTLMHIANQP----REKEFHVTILDIFALILKEQTAE
Swi1    187   ----------ETIISFAKRHILDLIVTLVSNLD----EFEHFDVYILEIVYNLIRGCKPS  232
Tof1          PNVSMDDISLNTVISSFHKNKVFGFLLTLTSSLSKEFDQDFINIPLLEIMFYFTKDVNQE

Timeless      QLAGVGQGRLAQERSADFAE--------------LEVLRQREMAEKKTRALQRGNRHSRF
Timeout       SLADASLQRSLSEKQRDQQE--------------LLAARRRERARRQARPPP--GRHSRF
Tim-1         DLATKSEEVSTAEQKKTEEE--------------FRKIIENHVVKETQKRKS----FSRF
Swi1    233   ALFS------DASLTNSQTE--------------LNSLLLKESTQNRYLKRNAHTRHNRF  272
Tof1          LLFPRQFETGTHSKVVNKNESSSANNIVTSAGFELSKLLQKEHQMRKNVIKHTSARHSRF

Timeless      GGSYIVQGLKSIGERD-LIFHKGLHNLRNYSSDLGKQPKKVPKRRQAARELSIQ------
Timeout       GGTYVIRNMKSVSDRD-VICHQALERVSSIDFDREKQQQKRS-HRHIQEEAQVT------
Tim-1         GGSYTIKGLKGISANSSQVVFKPIQNVEKHNFLDDRKAKKRAPRNRRPFEIDTNS-----
Swi1    273   GTMLSVQ----TEDRRFTIASQNIKTDGLDELDSHKRFRKRGTRRKHFDDINKS------  322
Tof1          GGLLSIQTPDKTRLTVSGSQALVDEKIALQKLDDSKKWNKRIIKKHQSVAAEGLPNSLLN

Timeless      ------RRSALNVRLFLRDFCSEFLENCYNRLMGSVKDHLLR-EKAQQHDETYYMWALAF
Timeout       ------RRSAFTVRLCLREYCIEVLRSAYNTLVRQVRRVLERNAGSSSHDDSYLLWAIRF
Tim-1         ------HFASSEVRGMLRDMVIRIIETCFNRLMKSSKTTVFVQVQKTSQINYFFLIKFVL
Swi1    323   ------FFINTEAGTALRNFAVEFLEAGFNPLFQSLLKDLEREDPRVLPIHKMQLLYVQS  376
Tof1          SQTGKAIFFTESNGKHFKEFINNFIDSGFNILLHSVTNYFTTEQDRMVTLEQVEYLLFFA

Timeless      FMAFNRAASFRPGLVSETLSVRTFHFIEQNLTNYYEMMLTDR-KEAASWARR---MHLAL
Timeout       FMEFNRLSGLQLQLVSESLSVQCFHWVLTRMQHDMDMIVSDK-KQARLWAKR---LHVAL
Tim-1         RFVRLSRQDHLLERISECIGVEAFHENNVQLTEYVENATTLKGVEAKSHGLK---AQYAL
Swi1    377   FFLEFMRFSSKPKKTEEIYSNDYSFGLAASVFDQRALIMHNRLMVESFEMKQWSTFQASM  436
Tof1          WFVKYQLLRSKIDNSADIKQVS-------EALKEVTFILVSSLLRSAYDLKNWTVTHAGM

Timeless      KAYQELLATVNEMDLSPDE-AVRESSRIIKNNIFYVMEYRELFLALFRKFDERCQPRSFL
Timeout       KTFRELLQSLLALQKLKDDNNARALFDMLLNNVCYVLEYRETVLHLLMNYNEAHSTKVFL
Tim-1         GAYNELVLLHRYIYEHAKEENERKFAKRALEHIVNVEEYRELPIFIIKKFSSSVLSNNFL
Swi1    437   LSMTQLLFTLRSMTLCSSEIYQRIADNLLSN-IFYQEEILLLVYSALKHFKTQSFG--YL  493
Tof1          IAFNELLNLVSRTKAAQEE--DSTDIEFIVSRLFSDERIQLLSNLPKIG---SKYSLQFM

Timeless      RDLVETTHLFLKMLERFCRSRGNLVVQNKQKKRRKKKKKVLDQAIVSGNVP-SSPEEVEA
Timeout       RDVVETANVFIKMMERFCQDSVVVQDKKRGRGASRKKKQAATKSKPPAAPQ-PTEEELSS
Tim-1         RELVLTTHHYMKLVERFVKTGALKKVTKKVKVRKATKKSKMSEEDVRSEFDGMSKKDLDR
Swi1    494   DAITELTIVLLKELEKFSSAKQYLYVKKRRRNQKSVDSNVLE-----------SDEDEES  542
Tof1          KSCIELTHSVLKVLEQYSDDKTLVIE---GKSRRQKKFNISEGDITKLIEEENVDRDEAL

Timeless      VWPALAEQLQCCAQNSELSMDS---VVPFDAASEVPVEEQRAEAMVRIQDCLLAGQAPQA
Timeout       KWAELATEVCSLLS-TELEMPEDEQPLPFDAASEKSIDDQREDCMIRINKLLRSEKLDQA
Tim-1         LWEESKGLVLQILKKEVPEMRG---MNPIDSQLDVPVDAQQKFAKLSIQRSLRSRGFPAA
Swi1    543   SLINAN------------------------------------------------------  548
Tof1          DILTSS------------------------------------------------------

Timeless      LTLLRSAREVWPE-----------------------------------------------
Timeout       IALLRAAREVWPE-----------------------------------------------
Tim-1         VGLYHASRALWPESFKRGLTDFQDSPGEEDQLQELEQLLKADMKKVAKDLKKAESCKTCD
Swi1          ------------------------------------------------------------
Tof1          ------------------------------------------------------------

Timeless      ------------------------------------------------------------
Timeout       ------------------------------------------------------------
Tim-1         EDPAYKKYDKMDATALQSLWEQSTDTLARILSHELPESESTSPVNWQLDITPDVQQKFAM
Swi1          ------------------------------------------------------------
Tof1          ------------------------------------------------------------
```

```
Timeless           -----------------------------GDVFGSQDISPEEEIQLLKQILSAPLPRQQG
Timeout            -----------------------------NEVFGAISAAPEDELLLLREIFMSNITTVES
Tim-1              LAIQRALRARDLPAAVGLYHTSRKLWPGDEAIFGAPGIGVEEEIAELKAILEADLHEVAR
Swi1               ------------------------------------------------------------
Tof1               ------------------------------------------------------------

Timeless           PEERGAEEEEEEEEEEEEE-------ELQVV---QVSEKEFNFLDYLKRFACSTVVRAYV
Timeout            EDKENEKDQKQDEYEDEDDDLDNEEYEEQDN---GLTEKTFKFDDFARRLLNPKIVRACT
Tim-1              EMKVAEDRAEDPDEEDPAEPYDSEQEEEEEVPAWKVEEIDFQFDSYVCKFSNVDVLKWYV
Swi1         549   ---------------------------------AAVEDRLFDFGRYESRYCDNGCIDSFV  575
Tof1               -----------------------------------LRSIEVNFQKVQANYMTEPVIETYI

Timeless           LLLRSYQQNSAHTNHCIVKMLHRLAHDLKMEALLFQLSVFCLFNRLLS--------DPAA
Timeout            LVLSDWADIPTRSLKAAVTILHRIAYGCKCAGMLFQAKLFRIFQQVFS--------VERD
Tim-1              FLLNDFSKNSTELNQALVKMLHRIAFDLKLPIKLYQVSLFQVFSKVNEHFTHLSKDLRKS
Swi1         576   LFLQCYQDLDSKQIHRAISFFYRIFVKQKCHVYLYRLDFLRVLDKMFN---DHVYFSTTN  632
Tof1               NFLERFRELEDDSIKKVFSFFHRVFVQAKEQALLFRFDLIILLREMLS----PDGLDRMS

Timeless           GAYKELVTFAKYILGKFFALAAVNQKAFVELLFWKNTAVVREMTEGY-------------
Timeout            VHQEELRRLAIFVVRKFVEVAPTNPKIYAELLFYKGIREANELESGY-------------
Tim-1              SRLYELYQFGFHLLKKFFSKFTG--DLAIEALFWKGPRECFEIENGY-------------
Swi1         633   SARQDFEQFFVYYMRKLSDALKDVPALFIELPFPKLTDTFYYLEYGK-------------  679
Tof1               RSRKYVSQFSDYFLARLKKRLKKSPAWFVGLLFPPLHNSEVGFYQRYGEYNVLNNESMYA

Timeless           GSLDDRSSSRRAPTWSPEEEAHLQELYLAN--KDVEGQDVVEAILAHLNTVPRTRKQIIH
Timeout            CDAYEAGTKG---AWTEEQESELRFLFEENQRNPETDKDVIDWILDNLVDKTRNRRTVLK
Tim-1              GSWVKSREADIR-VWTEDLEIELRNLYEEYRTMETRDGIDVLDFIEHNLSRARSRKKVAK
Swi1         680   SPLFSIHGSRKGPLYET-----------------VPGLSHLEKVAAVVACLINENKSDLL  722
Tof1               APASQFKPIPDEEALPPSILLDMKYGVLVSTLLDDGKTELLDQLLKHITHTLDIFKSWLT

Timeless           HLVQMG---LADSVKDFQRK---GTHIVLWTGDQE---LELQRLFEEFRDSDDVLGHIMK
Timeout            KLKELGLLFKAPTKRSTKSA---QSGKNVWQPEED---DELRSLYDQHRIEPDCLERLVN
Tim-1              KLIEFGFDLLGAKWKNSDKA---RMDSVLPIGDIQKWYDEWKEAGARGDLVNVLQEKLNE
Swi1         723   DELKVQLNCLISERKLITLA---DENKYINEGGND------------GERMGKNLKGDTD  767
Tof1               VNVNAGKETVNPPNEYFTLTGVLNNDPIFKDKDYRALLLLIGYSIPRKINEPCFLPGTVE

Timeless           NITAKRSRARIVDKLLALGLVAERRE----LYKKR----QKKLASSILPNGAESLKDFCQ
Timeout            EFAERRSKQQIIKRMLQIHLIADKSE----ILPAKKGRGKDKPKKDVEMEGEGDEFDFAE
Tim-1              DLGMEISRKKILKQLAHMDILYEKPKKEKPLPQWDTGLIEELKKLKEQYDDIPDALNMLG
Swi1         768   SFNTALLKDGKFRLLLELCGFEESDN-----------------NIDVQALWKLPNSVIID  810
Tof1               VSDLTVSCELVKKYLSTPFETPNGLPSSSYLLRVRSEKDSFSHNEQDGWEGDDDYDYNDP

Timeless           EDLEEEENLPEEDSEEEEEGGSEAEQVQGSLVLS-----NENLGQSLHQEGFSIPLLWLQ
Timeout            EPMFEDQGYKKPKSKPKKVQKRQMVRTPLDVGTI-----RALIGQVDSEK-YQSAIEWLQ
Tim-1              VNIVRYVMKRLSEKKPTRQVERHLESLGATIPERS---KKSEKNGKKFDDFLNDDDDDSE
Swi1         811   ELVEHAMLLRRFTDDPPTFEGTKPEDLLVRKQRG---------------------NVRLP  849
Tof1               YIVPDDQILSKSDAAYFKDLDNNASDKLKGTKFSKGIARSKKKDKRKRRKGEAKTNLPMF

Timeless           NCLIRAADDREEDG-CSQAVPLVPLTEENEEAME------NEQFQQLLRKLGVRPPASGQ
Timeout            ECLQDASEDTEEAVEDDDGVPLLPLMENQKNAME------DGDFQKVLVALGMQPPISGM
Tim-1              NDVGGGSEDDEEEEIVMKSKRIIPDSEDEEEHIE------QEEAQKKLEKVAEKP-----
Swi1         850   SSSEGETSDEEIEFEADDPITFANRREALNKITD------RKRKKMKTN-----------  892
Tof1               GDQDDERPQTVRERHGVFSKEFISDSEDDEDLMNPIFFENETYMRWLLDKNNGQLTEDRY

Timeless           ETFWRIPAKLSPTQLRRAAASLSQPEEEQKLQPELQPKVPGEQGSD--------EEHCKE
Timeout            EAYWRIPIYLNSADLILRSKILAGEEVDAEPEDEAADDEDGEEAEESDEEEDFLEKHSRQ
Tim-1              NTLMGMIAGRKRKLAQLESDSSDESDDDDSAEKEEKKLPAAEDDSD-------LEEDAVI
Swi1         893   ETIIDHTTRKKKENHLRSAKYIVDSDDDSETDIAFFQSEAALREKN--------------  938
Tof1               IQFAKFAAERMNNGGVVTGDYTSLFGGSIPSIESIRATESSSFAPDKSLISLASHVASEM

Timeless           HR----------------------------------------------------------
Timeout            RKENIATQQQRKLDSLMFNQSDDETEQRAPPKAKDKPKASEKKAKRKVKGKAKESKDCND
Tim-1              YK----------------------------------------------------------
Swi1               ------------------------------------------------------------
Tof1               SIFDVN------------------------------------------------------

Timeless           -----------------------------------------------------AQALRAL
Timeout            DGTDKAELEKPNTDDLFNQLRAKRATKIKLSDMALNESTNAAQEADDDAFNFNSEDYRAR
Tim-1              ------------------------------------------------------RSYVDA
Swi1         939   -----------------------------------------------------AQKASAL  945
Tof1               --------------------------------------------------NNNNNQLSDD

Timeless           LLAHKKKAGLASPEEEDAVGKEPLKAAPKKRQ----LLDSDEEQEED-------------
Timeout            LLELEDEEQNSKDADENGGDKENITNKSLQRARRVNVIDSDSDNDDDGALPADKTNKRPR
Tim-1              LLTGGSIAGNGITETRRDTSEE-----------------REDDDDED-------------
Swi1         946   FKRIDDLEMEGKLQEIEQLSEN-----------------SSSD-----------------  971
Tof1               DVNSESRNSLGSSQPSNSQNMFQSEVYSRKESTKRSLEASAADESDE-------------

Timeless           ------------------------EGRNRAPELGAPGIQKKKRYQIEDDEDD--
Timeout            SDDDGDSGHMDANAEEDSDEEANFLARKKPAKVDGEPAKRRRLAIIDDDDEDDF
Tim-1              -------------------------------PFTKKLTFKRRIVMSDNEDEA--
Swi1               ------------------------------------------------------
Tof1               -----------------------------DEEAIRLFGKKSRVVLSQGDSDD--
```
